# Sexual aggression by intruders in hooded crow *Corvus cornix*

**DOI:** 10.1007/s10211-015-0222-z

**Published:** 2015-09-16

**Authors:** Piotr Zduniak, Jakub Z. Kosicki, Reuven Yosef

**Affiliations:** Department of Avian Biology & Ecology, Faculty of Biology, Adam Mickiewicz University, Umultowska 89, 61-614 Poznań, Poland; P. O. Box 272, 88000 Eilat, Israel

**Keywords:** Corvids, Floaters, Sexual aggression, Social organization, Wetlands

## Abstract

The hooded crow *Corvus cornix* is a west Palaearctic, solitary nesting, monogamous corvid. In the breeding season, populations are characterized by a social organization wherein breeding pairs are territorial and non-breeding individuals, called floaters, live in flocks. During a study of the breeding ecology of the hooded crow, conducted in a protected flooded area, we monitored nests with video cameras. We recorded two separate incidents when intruders attacked a female at the nest. We believe that she remained in the nest in order to prevent the strangers cannibalizing the nestlings by mantling over the brood. The spatio-temporal occurrence of these attacks suggests that the observed behaviour is intraspecific sexual aggression wherein non-breeding males mounted an immobilized female.

**Electronic supplementary material:**

The online version of this article (doi:10.1007/s10211-015-0222-z) contains supplementary material, which is available to authorized users.

## Introduction

Intraspecific aggression is a widespread phenomenon across the animal kingdom. It often results in settling status, precedence or access to some object or space (Hinde 1970). It takes a variety of forms that range from physical conflicts to sexual violence. One of these is sexual aggression by forced copulation, being an extreme manifestation of intersexual conflict (Low 2004) and has been reported in a wide variety of animal species (Thornhill and Palmer 2000; Low 2005; Adler 2010). Among birds, it is mainly known from waterfowl (McKinney et al. 1983; Gowaty and Buschhaus 1998; Adler 2010). Forced copulations are recognized as a tactic within a male reproductive strategy (Low 2004; Adler 2010). Therefore, the occurrence of such behaviour is related to the social organization and mating system prevailing in the species. For example in socially monogamous species of waterfowl, with a generally low frequency of social polygyny, the only way of increasing the reproductive success for an unpaired male is by resorting to forced copulations (Adler 2010).

The hooded crow *Corvus cornix* is a west Palaearctic, socially monogamous, solitary nesting species belonging to the *Corvidae* family (Goodwin 1976), which is considered the most intelligent group of birds with cognitive capabilities comparable to apes (e.g., Emery and Clayton 2004; Emery 2006; Emery et al. 2007). The social organization of the hooded crow populations during the breeding season is based on their breeding status (Loman 1985; Saino and De Bernardi 1994) and is split into monogamous breeding pairs with well-defined territories and non-breeding individuals that live year-round in non-territorial flocks a.k.a., floaters (e.g., Newton 1998; Baglione et al. 2005; Penteriani et al. 2011). The presence of floaters in a population increases the competition for a common food source or increases the probability of extra-pair copulations with paired individuals (Lenda et al. 2012). Also, floaters increase the risk of cannibalism of nestlings, which can become an important limiting factor of the breeding success in crow populations (e.g., Wittenberg 1968; Yom-Tov 1974; Tompa 1975).

During our study of the breeding ecology of the hooded crow, we monitored several nests with surveillance cameras. Video tapings showed that on two separate occasions, intruders attacked the female at one of the nests. Our aim is to describe this rarely observed behaviour and propose an explanation in the context of the species’ social organization. Despite the fact that the hooded crow is a very widespread species in the west Palearctic, intraspecific social interactions are poorly studied and practically unknown from undisturbed and natural biotopes.

## Materials and methods

The study was conducted in the Warta Mouth National Park (N 52° 34′, E 14° 43′) in western Poland during the breeding season (April–June) in the year 2011. The reserve is located at the confluence of the Warta and the Odra river; it is a RAMSAR site (Convention on Wetlands of International Importance) and a wildfowl refuge of international importance (Grimmet and Jones 1989). The study area is largely undisturbed by humans because it is inaccessible by foot and water-logged throughout the year. This allowed us to study the behaviour of a primeval hooded crow population that is unmodified by human contact, unlike most other corvid studies, which were conducted in agricultural, rural or urban areas. The breeding population is characterized by a relatively high and stable density that varies from 2.6 to 3.8 pairs/km^2^ and breeding performance is affected by water level (Zduniak 2009, 2010).

This study is part of a larger project conducted in the western part of the reserve for 10 years, in an area of ca. 16 km^2^. The standard research methods of nest finding and visits are described in Zduniak (2010). Furthermore, we monitored six nests continuously for 18 h a day with video cameras for the 32 days of the nestling stage, from hatching to fledgling. The monitored nests were scattered unevenly across the study area. In order to avoid disturbance to the breeding pairs, the cameras were wrapped in willow (*Salix* spp.) bark and connected with a digital recorder concealed 6–14 m below the nest. Batteries were changed every 2–3 days. Cameras were attached to trees 1.5–2.0 m above the nests and focused so as to document activity on the nest.

We documented two separate, unusual events that occurred on 10 May at 20.43 h (movie 1—64.5 s) and on 12 May at 20.06 h (movie 2—31.9 s) at a nest with three nestlings at the age of 9 and 11 days, respectively. At this stage, the nestlings still have ca. 22 days before fledgling and both parents feed the brood.

The individuals observed in the movies could be individually identified as the breeding female and her mate on the basis of their plumage patterns on the back known from the daily monitoring of the nest. In addition, three previously undocumented individuals (thereafter intruders) sequentially arrived at the nest and attacked the female. Only one of these was individually identified in both the movies on the basis of a small dark spot on the back.

In order to describe the movies and to quantify the behaviours observed, we applied the Observer XT 11 software (Noldus Information Technology, Wageningen, The Netherlands). In the description, the time lapse since the beginning of each movie is presented in seconds and given in parentheses.

## Results

**Fig. 1.**
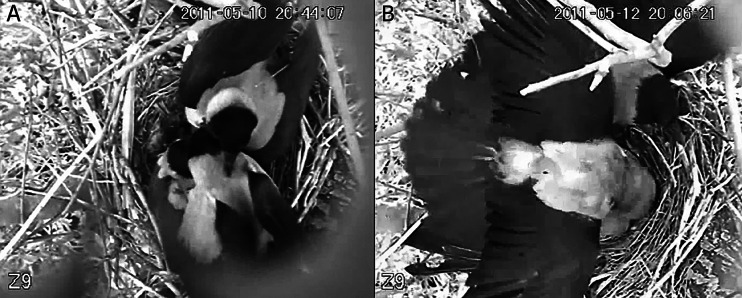
Sexual aggression of intruders that attacked the female at the nest; **a** first event, **b** second event

The first event (Fig. 1a; movie 1) lasted 59.4 s. It is evident that the female has noticed an approaching intruder 4 s after the video starts and moved from the rim of the nest to mantle over the nestlings with her body and slightly extended wings. After 2.1 s (6.1 s of the movie), an intruder appeared at the nest, and after 0.6 s (6.7 s) mounted the female. A second intruder arrived after 2.2 s (8.9 s) and tried to insinuate himself between the female and the first individual. After 10.0 s (18.9 s), the intruder pushed the first intruder aside and mounted the female. After 0.5 s (19.4 s), a third intruder arrived and landed first on the top of the two individuals, and after 4.9 s (24.3 s) moved to the left side of the nest and also attempted to access the female. In this way, all three individuals jostled each other to mount the female. After 19.7 s (39.1 s), the second intruder left the nest while the first and the third intruders continued to fight for access to the female for the next 3.8 s. Eventually (42.9 s), the first intruder mounted the female while the third intruder pushed and pecked at him for the next 15.3 s. The third intruder left the nest (58.2 s), while the first intruder remained on the female for the next 4.4 s and then left the nest (62.6 s). Almost immediately, after 0.3 s, a fourth individual appeared (62.9 s) and stayed at the nest for 0.5 s, and flew away (63.4 s).

The second event (Fig. 1b, movie 2) lasted 26.2 s. In this movie also, right at the start, the female has noticed the approaching intruders and settled over the nestlings in the manner described before. After 5.8 s of the movie, the first intruder appeared at the nest and 0.7 s later (6.5 s) jumped on the female’s back. Simultaneously, a second individual arrived after 0.9 s (7.4 s) and the first male was supplanted by the second who mounted the female and the first male left. After 2.9 s (10.3 s) a third individual appeared (identified based on his plumage as the paired male) and landed on the copulating male and pecked it on the head for 14.2 s. At this juncture (21.0 s) another intruder appeared at the nest for 1.5 s and pecked the paired male on its head and flew away. Following this, the paired male moved to the right of the nest (24.5 s) and after 0.9 s (25.4 s), the second intruder flew from the nest followed by the paired male (26.4 s).

In both the described cases, all (three in movie 1, two in movie 2) intruders attempted to gain access to the female in order to copulate with her. In movie 2, the paired-male defended his mate and drove away the intruders. At the end of movie 1, the last individual behaved like the breeding male and chased away the intruders; however, this could not be determined conclusively because the bird was present for only 0.5 s, which did not allow positive identification. Both intrusions occurred in the evening, when a flock of about 50 non-breeding crows appeared regularly for nine consecutive days circled the area and roosted on two willows at a distance of ca. 350 m from the observed nest. No intrusion was recorded in the other five nests observed with the same intensity during the presence of the roosting flock (i.e. 18 h for 9 days), but which were located in a different part of the study area at distances of 2.6 to 5.7 km from the floater roost. The presence of the non-breeding flock in mid May in the study area was exceptional in comparison to the other 9 years of the study, when flocks of non-breeding birds usually appeared 4 weeks after the breeding season.

Subsequently, the breeding attempt was successful and all three nestlings fledged from the nest.

## Discussion

The observed behaviours suggest that the intruders attacked a paired female that did not escape even though she noticed their approach. The female raised her feathers but did not resist or struggle with the intruders. We assume that this was because she wished to protect her nestlings. If this is correct, then she was voluntarily immobilized on the nest and the arriving intruders could mount her easily. The intruders are most probably young males from the non-breeding flock observed in the vicinity of the monitored nest.

In general, most floaters in avian populations are young individuals and the sex ratio is male skewed (Newton 1998; Penteriani et al. 2011). In the case of the hooded crow, which is a relatively long-lived species, individuals attain sexual maturity at 2 years of age, although the majority does not establish a territory until the age of 3, and some even later (Loman 1985). In socially monogamous avian species, male testosterone level is elevated mainly during the first part of the breeding season, when territories are established and birds start to breed (Wingfield and Farner 1993; Vleck and Brown 1999). In our study, it is probable that in the case of a paired male, the testosterone level declines after the egg laying period and the unpaired males may be characterized by elevated testosterone for a longer period of time including the brooding period (cf. Wingfield et al. 1987; McGlothlin et al. 2007). Simultaneously, forced copulation behaviour can be positively associated with testosterone levels (Davis 2002). In the studied population, early May, when we recorded the behaviour, is the last chance for a reproductive attempt. Hence, we assume testosterone levels, and therefore the motivation to copulate, are still elevated in non-breeding males. Breeding females, who are immobilized on the nest to protect their nestlings, may be a target for forced copulations by unpaired males.

Presence of intruders at the nest also constitutes a hazard to well-developed nestlings, which in our specific case were three fairly large nestlings. This may be why the female did not flee the nest as the intruders approached but instead moved on top of the nestlings. However, from a close scrutiny of the footage, it appears unlikely that the intrusion into the active nest was motivated by an attempt to cannibalize the nestlings because the intruders did not try to get the nestlings or attempt to push the female from the nest. The nestlings were visible on the video in several instances, as they were not entirely covered by the female, and the intruders did not try to grab them.

Forced copulations are considered to be rare among birds except in waterfowl (Gowaty and Buschhaus 1998; Adler 2010). A passerine that is well studied in New Zealand and has a comparatively high rate of forced copulation is the stitchbird *Notiomystis cincta* (Low 2004, 2005). Further instances of such behaviour have been reported for 13 species (cf. review in Gowaty and Buschhaus 1998). Among corvids, forced copulations has been recorded in the colonially breeding Rook *C. frugilegus*, where similar to the present study, a multimale group attempted to mount females (Roskaft 1983), and in the cooperatively breeding American Crow *C. brachyrhynchos*, where extra pair copulation attempts were initiated by males, and mostly appeared to be resisted by females (Townsend 2009). Similarly, in this paper we describe a previously unreported and untypical aggressive social behaviour in the hooded crow.

## Electronic Supplementary Material

ESM 1 (MPG 14790 kb)

ESM 2 (MPG 10700 kb)
